# Supplementary material for: Space station biomining experiment demonstrates rare earth element extraction in microgravity and Mars gravity
Source: Nat Commun. 2020 Nov 10;11:5523. doi: 10.1038/s41467-020-19276-w (PMC7656455; doi:10.1038/s41467-020-19276-w)
Supplement: Supplementary file 1 — Supplementary Information [file 41467_2020_19276_MOESM1_ESM.pdf]

Supplementary information for

Space station biomining experiment demonstrates rare earth element extraction in microgravity and Mars gravity

by Cockell, Santomartino et al.

Supplementary Fig. 1. Bioleaching and non-biological controls leaching of medium-abundant Rare Earth Elements. Quantities (ng) of the medium-abundant Rare Earth Elements (REEs) in each of the chambers at the end of the experiment (described in the text) for each of the three organisms examined and non-biological controls. The three most (Ce, Nd, La) and least (Tm, Lu, Tb) abundant REEs are shown in Fig. 2. ○ show triplicate biologically independent measurements and the mean is given as ▷. Error bars represent standard deviations.

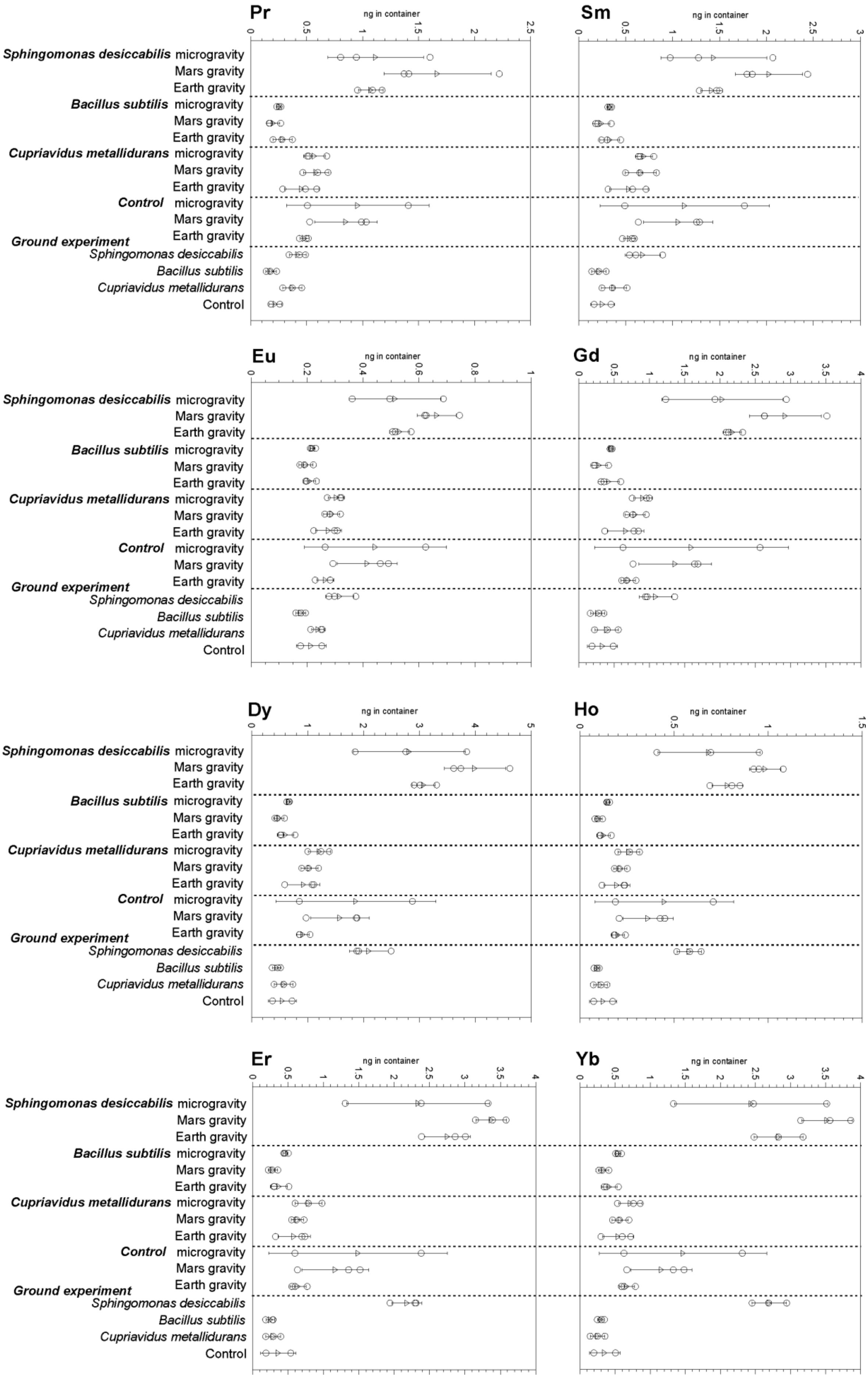

Pr
Sm
Eu
Gd
Dy
Ho
Er
Yb
ng in container
Sphingomonas desiccabilis microgravity
Mars gravity
Earth gravity
Bacillus subtilis microgravity
Mars gravity
Earth gravity
Cupriavidus metallidurans microgravity
Mars gravity
Earth gravity
Control microgravity
Mars gravity
Earth gravity
Ground experiment
Sphingomonas desiccabilis
Bacillus subtilis
Cupriavidus metallidurans
Control

Supplementary Fig. 2. Ground-based post-flight pH experiments investigating the pH during cultivation. Figure shows pH of the culture medium with the three microorganisms and the non-biological negative control, with (solid lines) and without (dotted lines) the presence of the basalt rock, during the main experimental timeframe and after NOTOXhisto fixation (day 21). The pH was measured twice on day 21, before and after fixative addition. Mean values (n=3, black dots) for each sample are plotted, and error bars (in red) indicate standard deviations of three biologically independent samples.

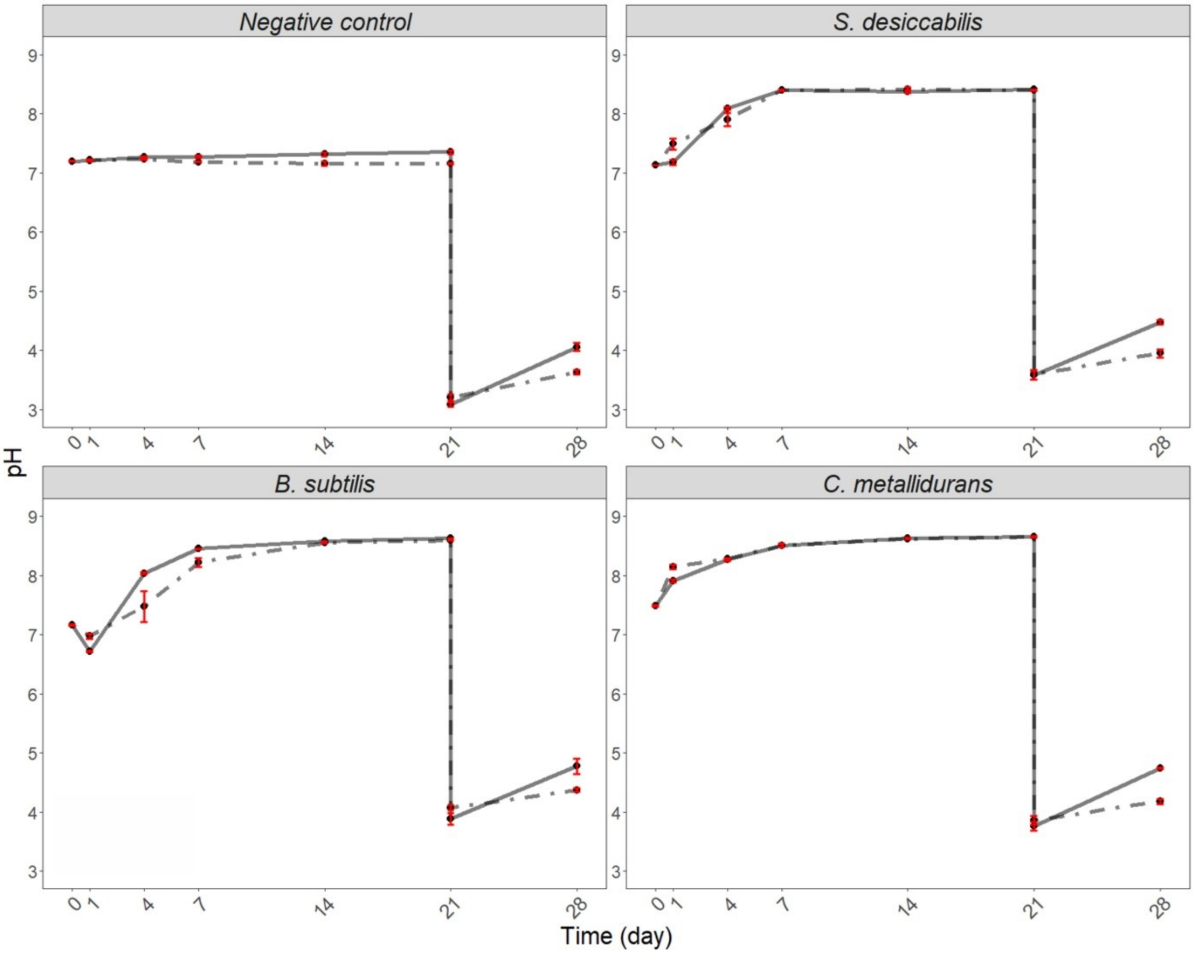

Supplementary Table 1. Abundance of major elements in basaltic rock (%) used for the BioRock experiment, reported in the standard form as equivalent oxide concentrations.

**Mineral**	**Element**	**%**
Na_2O	Sodium (Na)	1.92
MgO	Magnesium (Mg)	10.00
Al_2O_3	Aluminum (Al)	15.35
SiO_2	Silicon (Si)	47.48
P_2O_5	Phosphorus (P)	0.128
K_2O	Potassium (K)	0.162
CaO	Calcium (Ca)	11.69
TiO_2	Titanium (Ti)	1.344
MnO	Manganese (Mn)	0.186
Fe_2O_3	Iron (Fe)	12.12

Supplementary Table 2. Mean (± standard deviation) of quantities of total REEs (ng) in the chamber fluid (6 mL) at the end of the experiment shown to two decimal places. All data points shown in Fig. 2 and Supplementary Fig. 1. Mars gravity and Earth gravity refer to simulated Martian (0.4 x *g*) and terrestrial (1 x *g*) gravity on ISS.

**Organism/gravity**		**La**	**Ce**	**Pr**	**Nd**	**Sm**	**Eu**	**Gd**	**Tb**	**Dy**	**Ho**	**Er**	**Tm**	**Yb**	**Lu**
S. desiccabilis	**Microgravity**	3.60±1.26	8.85±2.89	1.12±0.43	5.35±2.02	1.44±0.57	0.51±0.16	2.03±0.86	0.42±0.14	2.82±1.00	0.69±0.27	2.34±1.01	0.42±0.16	2.44±1.09	0.49±0.20
**on ISS**	**Mars gravity**	4.96±0.51	9.26±1.94	1.67±0.48	7.89±1.99	2.03±0.36	0.66±0.07	2.93±0.51	0.57±0.08	3.99±0.55	0.98±0.08	3.37±0.22	0.58±0.04	3.52±0.36	0.68±0.08
	**Earth gravity**	3.74±0.51	7.18±0.99	1.07±0.11	5.20±0.47	1.42±0.12	0.53±0.04	2.18±0.13	0.44±0.01	3.08±0.21	0.78±0.08	2.75±0.32	0.49±0.06	2.83±0.35	0.57±0.07
B. subtilis	**Microgravity**	0.80±0.04	2.83±0.59	0.25±0.02	1.27±0.07	0.33±0.02	0.22±0.01	0.45±0.01	0.13±0.00	0.66±0.03	0.15±0.01	0.47±0.03	0.14±0.01	0.55±0.04	0.17±0.01
**on ISS**	**Mars gravity**	0.61±0.13	2.32±0.19	0.20±0.06	0.98±0.28	0.24±0.09	0.20±0.02	0.28±0.12	0.11±0.02	0.49±0.09	0.10±0.02	0.28±0.07	0.11±0.01	0.33±0.07	0.12±0.01
	**Earth gravity**	0.98±0.34	3.59±0.63	0.28±0.09	1.31±0.39	0.33±0.11	0.21±0.02	0.42±0.15	0.12±0.02	0.61±0.15	0.13±0.04	0.37±0.12	0.12±0.02	0.42±0.11	0.15±0.02
C. metallidurans	**Microgravity**	1.88±0.21	3.58±0.63	0.57±0.10	2.59±0.47	0.70±0.09	0.30±0.03	0.90±0.13	0.21±0.02	1.21±0.20	0.26±0.06	0.79±0.19	0.17±0.03	0.72±0.17	0.18±0.03
**on ISS**	**Mars gravity**	2.00±0.28	4.02±0.42	0.59±0.11	2.62±0.56	0.66±0.16	0.29±0.03	0.80±0.14	0.19±0.03	1.04±0.15	0.21±0.03	0.63±0.09	0.15±0.01	0.57±0.12	0.16±0.02
	**Earth gravity**	1.61±0.48	2.88±0.74	0.46±0.15	2.15±0.80	0.54±0.21	0.28±0.05	0.66±0.26	0.17±0.04	0.93±0.29	0.20±0.07	0.59±0.23	0.15±0.03	0.54±0.22	0.16±0.04
**Non-biological**	**Microgravity**	3.22±2.20	6.45±3.99	0.96±0.64	4.68±3.49	1.13±0.90	0.44±0.25	1.60±1.37	0.30±0.21	1.86±1.43	0.45±0.37	1.49±1.26	0.29±0.19	1.47±1.19	0.33±0.22
**control on ISS**	**Mars gravity**	2.56±0.89	5.79±2.06	0.85±0.28	4.28±1.46	1.06±0.37	0.42±0.11	1.36±0.52	0.26±0.07	1.58±0.52	0.36±0.13	1.17±0.47	0.24±0.07	1.16±0.44	0.27±0.08
	**Earth gravity**	1.66±0.23	4.39±1.26	0.48±0.04	2.28±0.24	0.54±0.07	0.27±0.03	0.70±0.10	0.16±0.02	0.92±0.11	0.20±0.03	0.64±0.11	0.16±0.01	0.67±0.11	0.18±0.02
**Ground 1 *g***	***S. desiccabilis***	1.20±0.24	3.65±0.19	0.42±0.07	2.20±0.51	0.68±0.19	0.32±0.05	1.09±0.23	0.28±0.05	2.10±0.35	0.58±0.06	2.18±0.21	0.43±0.04	2.70±0.25	0.57±0.06
**experiment**	***B. subtilis***	0.56±0.16	2.80±0.15	0.18±0.05	0.87±0.26	0.22±0.08	0.18±0.02	0.27±0.10	0.10±0.01	0.45±0.08	0.09±0.01	0.25±0.06	0.10±0.01	0.30±0.05	0.12±0.01
	C. metallidurans	1.30±0.26	3.63±0.15	0.37±0.08	1.61±0.44	0.37±0.13	0.24±0.02	0.39±0.17	0.12±0.03	0.57±0.17	0.11±0.04	0.29±0.10	0.10±0.02	0.25±0.10	0.10±0.02
	**Control (non-biology)**	0.84±0.22	3.81±1.99	0.23±0.06	1.09±0.35	0.27±0.13	0.13±0.05	0.44±0.21	0.06±0.03	0.47±0.25	0.12±0.07	0.43±0.25	0.06±0.03	0.40±0.22	0.07±0.04

Supplementary Table 3. Percent difference in mean (± standard deviation) leached concentration of REEs in biological experiments compared to experimental non-biological controls. Mars gravity and Earth gravity refer to simulated Martian (0.4 x *g*) and terrestrial (1 x *g*) gravity on the ISS. Ground experiment was performed at 1 x *g* (true Earth gravity).

**Organism/gravity**		**La**	**Ce**	**Pr**	**Nd**	**Sm**	**Eu**	**Gd**	**Tb**	**Dy**	**Ho**	**Er**	**Tm**	**Yb**	**Lu**
S. desiccabilis	**Microgravity**	111.9±85.8	137.2±96.0	116.6±89.4	114.2±95.6	127.4±113.3	115.9±75.9	127.4±122.2	137.7±103.6	151.2±12.8	153.1±139.8	157.0±149.3	148.1±112.2	166.0±153.8	146.0±115.6
**on ISS**	**Mars gravity**	193.6±69.9	159.8±66.0	196.3±85.8	184.4±78.4	191.2±74.3	159.8±44.5	214.6±89.7	215.5±67.0	253.2±90.7	272.5±103.6	288.7±117.8	241.8±71.1	303.5±117.9	249.6±77.2
	**Earth gravity**	225.5±44.0	163.6±52.0	222.3±29.9	228.7±31.8	261.6±38.6	200.3±26.9	311.9±49.1	268.2±27.0	335.3±46.8	387.3±76.5	429.2±92.0	308.0±45.5	423.0±85.3	311.9±49.8
B. subtilis	**Microgravity**	24.9±17.0	43.9±28.6	26.4±17.7	27.1±20.3	29.4±23.5	49.5±28.4	28.5±24.6	44.4±30.1	35.3±27.1	32.9±27.1	31.2±26.6	48.7±32.0	37.1±30.2	51.6±35.0
**on ISS**	**Mars gravity**	23.7±9.7	40.0±14.6	23.8±10.3	22.9±10.2	23.1±11.7	47.2±13.5	20.8±11.6	41.0±13.1	30.9±11.7	26.9±11.4	24.4±11.4	45.0±13.5	28.6±12.4	46.0±14.0
	**Earth gravity**	58.8±22.2	81.7±27.5	58.9±18.6	57.7±18.3	61.1±20.7	78.9±12.2	60.2±23.2	76.3±16.3	66.0±17.9	62.2±20.2	58.2±21.5	75.6±12.1	62.9±19.3	80.5±14.5
C. metallidurans	**Microgravity**	58.4±40.4	55.5±35.7	59.4±40.8	55.4±42.6	61.8±49.9	68.6±39.7	56.6±49.4	69.5±47.8	64.9±50.9	58.0±49.4	53.0±46.7	61.1±41.3	48.9±41.3	55.2±38.3
**on ISS**	**Mars gravity**	78.2±29.3	69.4±25.7	68.8±26.3	61.3±24.7	62.3±26.6	69.4±19.2	58.4±24.5	71.5±22.4	65.6±23.8	59.2±23.9	54.1±23.1	63.9±19.0	49.4±21.1	59.1±18.0
	**Earth gravity**	97.1±31.9	65.6±25.3	94.5±33.1	94.6±36.5	98.7±39.8	104.3±21.2	95.1±39.6	102.9±27.1	101.0±34.0	97.0±37.6	91.4±39.1	93.2±21.8	81.2±35.2	85.5±22.0
**Ground**	***S. desiccabilis***	157.8±54.6	96.0±50.6	191.0±59.6	208.5±84.1	267.6±151.7	147.4±43.7	326.0±219.6	258.0±88.2	382.4±184.1	473.3±282.4	601.6±416.0	378.5±119.5	767.4±482.4	440.1±155.3
**experiment**	***B. subtilis***	73.8±29.6	73.6±38.8	82.4±30.2	82.5±36.5	84.8±51.6	82.7±22.1	79.6±58.6	90.9±28.9	81.7±39.6	72.1±43.8	69.6±50.5	90.5±28.8	85.2±54.9	94.8±32.7
	C. metallidurans	171.0±59.3	95.6±50.3	167.6±57.7	152.1±65.5	147.0±89.8	111.3±29.7	117.7±90.5	111.5±40.6	103.9±56.1	88.0±59.3	80.2±62.1	89.4±30.6	71.2±53.4	80.6±29.6

Supplementary Table 4. Statistical (two-tailed Student's *t*-test) *p* values of differences between selected treatments shown in Supplementary Table 2. *P* values below 0.05 are shaded dark blue and those below 0.1 are shaded light green. 'Control' refers to non-biological control

	La	Ce	Pr	Nd	Sm	Eu	Gd	Tb	Dy	Ho	Er	Tm	Yb	Lu
***S. desiccabilis* on ISS**														
Microgravity (biology vs. control)	0.814	0.484	0.754	0.798	0.659	0.722	0.682	0.498	0.434	0.460	0.460	0.442	0.415	0.483
Mars gravity (biology vs. control)	0.015	0.102	0.063	0.065	0.031	0.028	0.020	0.008	0.005	0.002	0.002	0.002	0.002	0.003
Earth gravity (biology vs. control)	0.003	0.039	0.001	0.001	0.000	0.001	0.000	0.000	0.000	0.000	0.000	0.001	0.001	0.001
Microgravity biology vs. Mars gravity biology	0.159	0.851	0.211	0.196	0.202	0.222	0.197	0.166	0.149	0.143	0.158	0.184	0.178	0.199
Microgravity biology vs. Earth gravity biology	0.869	0.396	0.870	0.909	0.953	0.875	0.789	0.790	0.685	0.585	0.538	0.531	0.589	0.525
Mars biology vs. Earth gravity biology	0.043	0.174	0.104	0.086	0.049	0.043	0.069	0.043	0.053	0.041	0.051	0.097	0.074	0.147
***B. subtilis* on ISS**														
Microgravity (biology vs. control)	0.128	0.190	0.127	0.161	0.192	0.193	0.213	0.217	0.208	0.220	0.221	0.234	0.238	0.268
Mars gravity (biology vs. control)	0.020	0.044	0.017	0.019	0.020	0.026	0.024	0.024	0.024	0.028	0.032	0.030	0.031	0.033
Earth gravity (biology vs. control)	0.045	0.378	0.023	0.022	0.042	0.061	0.057	0.079	0.043	0.053	0.049	0.036	0.049	0.099
Microgravity biology vs. Mars gravity biology	0.067	0.223	0.219	0.158	0.177	0.201	0.064	0.052	0.036	0.016	0.014	0.013	0.012	0.008
Microgravity biology vs. Earth gravity biology	0.433	0.203	0.570	0.859	0.995	0.484	0.710	0.532	0.584	0.356	0.270	0.142	0.142	0.163
Mars biology vs. Earth gravity biology	0.156	0.029	0.241	0.296	0.338	0.525	0.282	0.376	0.300	0.289	0.330	0.297	0.311	0.184
***C. metallidurans* on ISS**														
Microgravity (biology vs. control)	0.335	0.275	0.340	0.347	0.434	0.379	0.413	0.462	0.457	0.414	0.379	0.351	0.326	0.304
Mars gravity (biology vs. control)	0.377	0.175	0.214	0.148	0.164	0.155	0.147	0.172	0.163	0.140	0.126	0.098	0.088	0.074
Earth gravity (biology vs. control)	0.884	0.148	0.789	0.810	0.959	0.737	0.841	0.860	0.963	0.896	0.727	0.622	0.421	0.329
Microgravity biology vs. Mars gravity biology	0.587	0.367	0.859	0.949	0.749	0.517	0.392	0.359	0.292	0.301	0.259	0.306	0.286	0.302
Microgravity biology vs. Earth gravity biology	0.426	0.281	0.345	0.456	0.277	0.419	0.224	0.204	0.235	0.285	0.298	0.360	0.330	0.382
Mars biology vs. Earth gravity biology	0.292	0.081	0.309	0.450	0.456	0.732	0.477	0.507	0.597	0.709	0.760	0.844	0.838	0.879
**Control comparisons on ISS**														
Microgravity control vs. Mars control	0.655	0.816	0.803	0.862	0.908	0.865	0.796	0.772	0.758	0.718	0.698	0.701	0.691	0.673
Microgravity control vs. Earth control	0.275	0.436	0.253	0.284	0.306	0.279	0.305	0.291	0.301	0.298	0.295	0.292	0.295	0.298
Mars control vs. Earth control	0.164	0.371	0.088	0.079	0.074	0.080	0.094	0.083	0.099	0.116	0.133	0.119	0.130	0.132
**Ground 1 g experiments**														
S. desiccabilis vs. control	0.128	0.895	0.049	0.073	0.070	0.119	0.036	0.027	0.013	0.005	0.003	0.002	0.002	0.003
B. subtilis vs. control	0.311	0.413	0.464	0.536	0.689	0.317	0.646	0.642	0.534	0.448	0.484	0.621	0.697	0.796
C. metallidurans vs. control	0.096	0.883	0.122	0.242	0.391	0.513	0.748	0.663	0.914	0.772	0.670	0.619	0.519	0.400
**Ground and ISS comparisons**														
S. desiccabilis ground vs. ISS Earth gravity	0.001	0.004	0.001	0.002	0.004	0.004	0.002	0.007	0.014	0.028	0.063	0.201	0.618	0.999
B. subtilis ground vs. ISS Earth gravity	0.129	0.103	0.148	0.177	0.200	0.116	0.209	0.186	0.175	0.161	0.196	0.180	0.158	0.142
C. metallidurans ground vs. ISS Earth gravity	0.380	0.161	0.451	0.360	0.320	0.275	0.204	0.181	0.140	0.120	0.111	0.086	0.105	0.087
Ground control vs. ISS Earth gravity control	0.022	0.704	0.009	0.018	0.041	0.261	0.075	0.079	0.097	0.178	0.173	0.124	0.108	0.145

Supplementary Table 5. Quantity of Rare Earth Elements leached in biological experiments as percentage of total concentration in basalt slide. Tables shown separately for *S. desiccabilis*, *B. subtilis* and *C. metallidurans* and ground controls. Tables show means and standard deviations (%). Mars gravity and Earth gravity refer to simulated Martian (0.4 x *g*) and terrestrial (1 x *g*) gravity on the ISS. The concentrations of REEs measured for each experiment were converted to percentages of concentrations in the basalt slide by assuming a mean basalt slide mass of 1.87 g.

	S. desiccabilis					
	**Microgravity mean**	**sd**	**Mars gravity mean**	**sd**	**Earth gravity mean**	**sd**
La	2.83E-02	9.90E-03	3.89E-02	3.98E-03	2.94E-02	4.02E-03
Ce	3.50E-02	1.14E-02	3.66E-02	7.67E-03	2.84E-02	3.90E-03
Pr	2.58E-02	9.84E-03	3.85E-02	1.10E-02	2.47E-02	2.55E-03
Nd	2.47E-02	9.35E-03	3.65E-02	9.19E-03	2.41E-02	2.19E-03
Sm	2.53E-02	9.94E-03	3.57E-02	6.29E-03	2.50E-02	2.04E-03
Eu	2.44E-02	7.75E-03	3.14E-02	3.33E-03	2.51E-02	1.68E-03
Gd	2.96E-02	1.25E-02	4.26E-02	7.44E-03	3.17E-02	1.84E-03
Tb	3.91E-02	1.28E-02	5.34E-02	7.19E-03	4.12E-02	7.30E-04
Dy	3.85E-02	1.37E-02	5.45E-02	7.46E-03	4.20E-02	2.85E-03
Ho	4.58E-02	1.82E-02	6.58E-02	5.61E-03	5.23E-02	5.50E-03
Er	5.13E-02	2.22E-02	7.39E-02	4.72E-03	6.03E-02	7.11E-03
Tm	7.82E-02	2.99E-02	1.07E-01	7.57E-03	9.07E-02	1.04E-02
Yb	6.18E-02	2.77E-02	8.93E-02	9.09E-03	7.17E-02	8.85E-03
Lu	8.38E-02	3.48E-02	1.17E-01	1.30E-02	9.86E-02	1.19E-02

	B. subtilis					
	**Microgravity mean**	**sd**	**Mars gravity mean**	**sd**	**Earth gravity mean**	**sd**
La	6.31E-03	2.75E-04	4.77E-03	1.03E-03	7.66E-03	2.68E-03
Ce	1.12E-02	2.32E-03	9.16E-03	7.49E-04	1.42E-02	2.48E-03
Pr	5.83E-03	4.28E-04	4.66E-03	1.32E-03	6.55E-03	1.99E-03
Nd	5.87E-03	3.42E-04	4.52E-03	1.30E-03	6.07E-03	1.81E-03
Sm	5.84E-03	3.43E-04	4.30E-03	1.59E-03	5.83E-03	1.84E-03
Eu	1.04E-02	4.69E-04	9.28E-03	1.17E-03	9.90E-03	1.00E-03
Gd	6.63E-03	2.03E-04	4.14E-03	1.69E-03	6.12E-03	2.19E-03
Tb	1.26E-02	5.29E-05	1.02E-02	1.53E-03	1.17E-02	2.23E-03
Dy	8.97E-03	3.56E-04	6.65E-03	1.24E-03	8.27E-03	2.00E-03
Ho	9.85E-03	5.48E-04	6.50E-03	1.34E-03	8.40E-03	2.34E-03
Er	1.02E-02	7.35E-04	6.24E-03	1.47E-03	8.17E-03	2.64E-03
Tm	2.57E-02	1.42E-03	1.99E-02	1.87E-03	2.23E-02	2.92E-03
Yb	1.38E-02	1.06E-03	8.42E-03	1.84E-03	1.07E-02	2.80E-03
Lu	2.96E-02	2.04E-03	2.15E-02	2.04E-03	2.54E-02	3.71E-03

	C. metallidurans					
	**Microgravity mean**	**sd**	**Mars gravity mean**	**sd**	**Earth gravity mean**	**sd**
La	1.48E-02	1.69E-03	1.57E-02	2.23E-03	1.27E-02	3.77E-03
Ce	1.42E-02	2.49E-03	1.59E-02	1.65E-03	1.14E-02	2.93E-03
Pr	1.31E-02	2.24E-03	1.35E-02	2.61E-03	1.05E-02	3.57E-03
Nd	1.20E-02	2.19E-03	1.21E-02	2.59E-03	9.95E-03	3.70E-03
Sm	1.23E-02	1.58E-03	1.16E-02	2.90E-03	9.42E-03	3.61E-03
Eu	1.44E-02	1.34E-03	1.36E-02	1.36E-03	1.31E-02	2.17E-03
Gd	1.32E-02	1.88E-03	1.16E-02	2.08E-03	9.67E-03	3.77E-03
Tb	1.98E-02	2.31E-03	1.77E-02	2.45E-03	1.58E-02	3.86E-03
Dy	1.65E-02	2.67E-03	1.41E-02	2.09E-03	1.26E-02	3.97E-03
Ho	1.74E-02	3.86E-03	1.43E-02	2.29E-03	1.31E-02	4.58E-03
Er	1.73E-02	4.15E-03	1.39E-02	1.92E-03	1.28E-02	4.99E-03
Tm	3.22E-02	5.46E-03	2.82E-02	2.34E-03	2.75E-02	5.88E-03
Yb	1.82E-02	4.23E-03	1.45E-02	2.95E-03	1.38E-02	5.55E-03
Lu	3.17E-02	5.25E-03	2.77E-02	2.65E-03	2.70E-02	6.34E-03

	**Ground experiment**					
	***S. desiccabilis* mean**	**sd**	***B. subtilis* mean**	**sd**	***C. metallidurans* mean**	**sd**
La	9.43E-03	1.87E-03	4.41E-03	1.25E-03	1.02E-02	2.04E-03
Ce	1.44E-02	7.41E-04	1.11E-02	6.11E-04	1.44E-02	5.85E-04
Pr	9.75E-03	1.71E-03	4.21E-03	1.09E-03	8.56E-03	1.95E-03
Nd	1.02E-02	2.35E-03	4.03E-03	1.18E-03	7.42E-03	2.05E-03
Sm	1.20E-02	3.31E-03	3.81E-03	1.34E-03	6.60E-03	2.35E-03
Eu	1.50E-02	2.40E-03	8.40E-03	8.19E-04	1.13E-02	1.07E-03
Gd	1.59E-02	3.40E-03	3.87E-03	1.42E-03	5.73E-03	2.45E-03
Tb	2.66E-02	4.92E-03	9.36E-03	1.28E-03	1.15E-02	2.56E-03
Dy	2.86E-02	4.73E-03	6.11E-03	1.06E-03	7.77E-03	2.30E-03
Ho	3.88E-02	4.32E-03	5.90E-03	9.29E-04	7.21E-03	2.40E-03
Er	4.78E-02	4.57E-03	5.53E-03	1.32E-03	6.38E-03	2.30E-03
Tm	7.98E-02	6.71E-03	1.91E-02	1.78E-03	1.89E-02	2.93E-03
Yb	6.83E-02	6.24E-03	7.58E-03	1.29E-03	6.34E-03	2.65E-03
Lu	9.85E-02	1.02E-02	2.12E-02	1.51E-03	1.81E-02	2.63E-03

Supplemental Table 6. Rare Earth Element (REE) in cell pellets. Quantity of REEs in cell pellet as a percentage (%) of the total REEs in the pellet and bulk chamber fluid calculated from REE masses. Mars gravity and Earth gravity refer to simulated Martian (0.4 x *g*) and terrestrial (1 x *g*) gravity on the ISS. Values are shown as mean ± standard deviation. Means above 5 % are shaded in grey.

Organism/gravity		La	Ce	Pr	Nd	Sm	Eu	Gd	Tb	Dy	Ho	Er	Tm	Yb	Lu
S. desiccabilis	**Microgravity**	1.61±0.44	1.70±0.46	1.23±0.43	1.33±0.45	1.19±0.42	4.81±0.86	1.21±0.59	0.97±0.41	1.09±0.46	0.97±0.36	1.14±0.41	0.84±0.27	1.57±0.44	1.45±0.21
**on ISS**	**Mars gravity**	2.57±0.66	5.68±5.58	1.83±0.43	1.93±0.42	1.79±0.43	4.90±1.25	1.69±0.30	1.42±0.33	1.47±0.35	1.49±0.41	1.51±0.39	1.28±0.32	1.86±0.44	2.07±0.36
	**Earth gravity**	1.59±0.89	1.75±0.23	1.17±0.59	1.33±0.60	0.98±0.36	7.00±0.78	0.96±0.15	0.71±0.14	0.87±0.08	0.88±0.07	0.91±0.08	0.72±0.03	1.36±0.19	1.48±0.03
B. subtilis	**Microgravity**	2.33±0.59	8.06±9.03	1.28±0.28	1.59±0.44	1.53±0.60	15.54±4.08	1.07±0.33	0.27±0.20	0.67±0.32	0.76±0.45	1.11±0.08	0.18±0.19	0.92±0.37	0.47±0.21
**on ISS**	**Mars gravity**	6.42±0.68	4.28±0.46	3.16±0.50	3.59±0.36	2.41±0.34	19.00±4.63	2.98±0.58	0.61±0.34	1.02±0.57	1.48±0.59	2.13±1.05	0.35±0.49	2.11±1.23	0.54±0.59
	**Earth gravity**	19.99±14.55	34.34±22.02	14.93±12.87	13.24±10.75	10.65±10.59	18.16±6.10	11.26±8.83	4.31±3.85	5.87±4.88	6.02±4.67	6.36±3.93	2.22±1.89	4.81±3.08	2.16±1.43
C. metallidurans	**Microgravity**	3.18±1.97	4.32±1.98	3.25±3.07	2.20±0.94	2.10±0.79	7.86±3.40	1.62±0.50	0.76±0.10	1.18±0.29	1.02±0.19	1.29±0.33	0.39±0.26	1.31±0.62	0.86±0.59
**on ISS**	**Mars gravity**	2.46±1.50	10.78±9.55	1.93±1.50	1.93±1.27	1.90±1.35	8.07±4.25	2.09±1.46	1.11±0.77	1.42±1.10	1.61±1.26	1.81±1.38	0.70±0.71	2.06±1.60	1.17±0.99
	**Earth gravity**	4.34±2.74	5.34±2.58	2.63±1.82	2.81±1.60	2.84±1.47	13.79±5.64	2.31±1.60	1.12±1.29	1.40±1.16	1.49±1.28	1.89±1.05	0.56±0.67	1.68±1.25	1.18±1.05
**Ground 1 *g***	***S. desiccabilis***	2.35±1.05	6.66±7.68	1.40±0.79	1.81±0.91	1.63±0.69	7.94±3.55	1.60±0.93	0.84±0.64	1.12±0.58	1.29±0.70	1.35±0.65	0.96±0.46	1.64±0.67	1.85±0.66
**experiment**	***B. subtilis***	5.41±0.72	11.98±15.02	2.72±1.27	3.10±1.15	2.71±0.82	12.84±5.84	3.63±0.49	0.61±0.37	1.07±1.00	1.43±0.91	2.33±0.55	0.39±0.47	1.76±0.66	0.89±0.14
	C. metallidurans	3.76±1.46	3.24±0.68	2.48±1.22	2.97±1.27	3.98±1.82	22.36±9.82	3.77±2.08	1.35±0.92	1.82±0.93	2.59±1.57	2.98±1.59	0.62±0.75	2.57±2.00	0.87±0.79

Supplemental Table 7. Final pH values in the different EU solutions. Solutions were measured at the end of the experiment after sample fixation and return to Earth. The two contaminated control samples are shaded in grey.

**Organism/gravity**		**Sample 1**	**Sample 2**	**Sample 3**	**Mean**	**sd**
S. desiccabilis	**Microgravity**	4.31	4.31	4.28	4.30	0.02
**on ISS**	**Mars gravity**	4.31	4.31	4.25	4.29	0.03
	**Earth gravity**	4.24	4.32	4.29	4.28	0.04
B. subtilis	**Microgravity**	6.13	6.11	6.11	6.12	0.01
**on ISS**	**Mars gravity**	6.09	5.93	6.01	6.01	0.08
	**Earth gravity**	6.10	6.06	6.16	6.11	0.05
C. metallidurans	**Microgravity**	6.36	4.90	5.90	5.72	0.75
**on ISS**	**Mars gravity**	4.95	5.98	5.91	5.61	0.58
	**Earth gravity**	5.98	4.81	6.02	5.60	0.69
**Non-biological control**	**Microgravity**	4.18	4.24	4.42	4.30	0.17
**on ISS**	**Mars gravity**	4.28	4.35	4.22	4.28	0.07
	**Earth gravity**	4.29	4.46	4.26	4.34	0.11
**Ground 1 *g* experiment**	***S. desiccabilis***	4.28	4.37	4.33	4.33	0.05
	B. subtilis	6.01	6.11	5.70	5.94	0.21
	C. metallidurans	5.21	6.11	5.30	5.54	0.50
	**Control (non-biology)**	4.30	4.02	5.87	4.16	0.20

Supplementary Table 8. Acceleration (gravity) measurements. Values measured during the experiment on the ISS in each of the Experimental Units. The *g* level was measured every ten minutes for the duration of the active experimental phase in KUBIK. Values are shown as mean and standard deviation of these collected measurements.

Condition	EU No.	Contents of EU	*g* acceleration measured (mean ± sd)
Microgravity	1	*S. desiccabilis* x 2	0.059±0.011
Microgravity	2	*B. subtilis* and control	0.054±0.028
Microgravity	3	*C. metallidurans* x 2	0.033±0.020
Microgravity	10	*S. desiccabilis* and control	0.064±0.036
Microgravity	11	*B. subtilis* x 2	0.043±0.024
Microgravity	12	*C. metallidurans* and control	0.053±0.024
Mars gravity	13	*S. desiccabilis* x 2	0.422±0.018
Mars gravity	14	*S. desiccabilis* and control	0.411±0.016
Mars gravity	15	*B. subtilis* x 2	0.461±0.017
Mars gravity	16	*B. subtilis* and control	0.437±0.017
Mars gravity	17	*C. metallidurans* x 2	0.447±0.018
Mars gravity	18	*C. metallidurans* and control	0.389±0.018
Earth gravity	4	*S. desiccabilis* x 2	0.990±0.028
Earth gravity	5	*S. desiccabilis* and control	1.047±0.024
Earth gravity	6	*B. subtilis* x 2	1.039±0.036
Earth gravity	7	*B. subtilis* and control	1.032±0.036
Earth gravity	8	*C. metallidurans* x 2	1.036±0.027
Earth gravity	9	*C. metallidurans* and control	1.027±0.025

Supplementary Table 9. Final cell numbers in the culture chambers. Determined by direct microscopic enumeration of cell numbers as described in ref 31. Cell numbers shown as cells/mL (mean ± sd).

**Organism/gravity**		**Cell numbers**
S. desiccabilis	**Microgravity**	$(3.63 \pm 2.05) \times 10^9$
**on ISS**	**Mars gravity**	$(1.32 \pm 1.17) \times 10^9$
	**Earth gravity**	$(1.84 \pm 0.59) \times 10^9$
B. subtilis	**Microgravity**	$(5.38 \pm 2.69) \times 10^7$
**on ISS**	**Mars gravity**	$(5.75 \pm 2.07) \times 10^7$
	**Earth gravity**	$(3.32 \pm 0.50) \times 10^7$
C. metallidurans	**Microgravity**	$(1.01 \pm 0.71) \times 10^9$
**on ISS**	**Mars gravity**	$(7.26 \pm 7.19) \times 10^8$
	**Earth gravity**	$(2.33 \pm 1.90) \times 10^9$
**Ground 1 x *g* experiment**	***S. desiccabilis***	$(6.87 \pm 0.40) \times 10^8$
	B. subtilis	$(8.27 \pm 1.13) \times 10^7$
	C. metallidurans	$(1.83 \pm 0.02) \times 10^9$
